# Pictorial essay: Complications of a swallowed fish bone

**DOI:** 10.4103/0971-3026.76061

**Published:** 2011

**Authors:** Girish Bathla, Lynette LS Teo, Sunita Dhanda

**Affiliations:** Department of Diagnostic Imaging, National University Hospital 5, Lower Kent Ridge Road, Singapore

**Keywords:** Abscess, bowel, fishbone, perforation

## Abstract

Unintentional ingestion of a fishbone (FB) is common, especially in populations with a high consumption of seafood. In most instances, the ingested FB passes uneventfully through the gastrointestinal (GI) tract, usually within a week. However, in certain cases, the FB may become impacted and lead to complications. Awareness of these complications is important as patients usually present with nonspecific symptoms and could be unaware of having ingested an FB.

## Introduction

Unintentional ingestion of a fishbone (FB) is a relatively common clinical problem, especially in populations where unfilleted fish is a delicacy.[1] Fortunately, most of these FBs pass through the gastrointestinal (GI) tract without causing any serious complications. Problems arise when the ingested FBs become impacted at various sites within the GI tract. Owing to their sharp, pointed edges, FBs may rarely extend outside the GI tract and involve the surrounding organs, at times resulting in bizarre manifestations and clinical symptoms.

## Discussion

The most important risk factor for FB ingestion is the use of dentures, which can be implicated in up to 80% of the cases. Dentures are believed to impair palatal sensory feedback, which otherwise provides a protective mechanism for identifying sharp and hard-textured items in a food bolus.[1,2] Other less-established risk factors for accidental FB ingestion include rapid eating, extremes of age, alcohol abuse and mental retardation.[1,3,4]

### Upper aerodigestive tract and neck

Once swallowed, an FB may lodge itself in the upper aerodigestive tract, esophagus, stomach, small bowel or colon. It may rarely enter the airways and lodge itself within the trachea or major bronchi. Within the GI tract, entrapment in the upper aerodigestive tract is the most common complication following ingestion of an FB.[5,6] The most common site of impaction is usually at the level of the tonsils, although the impacted bone may be found at the base of the tongue, the vallecula or the pyriform fossa.[7] Because it is usually an acute event and presents with a short history, it is easily recognized and treated by the otolaryngologist.[5]

**Figure 1 (A,B):**
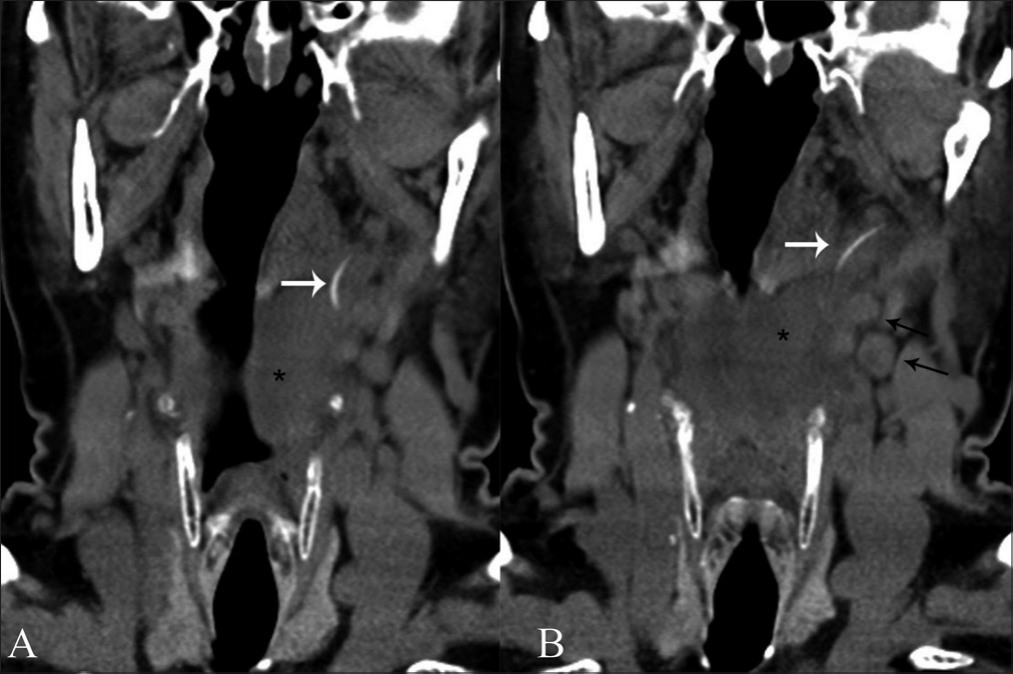
Noncontrast coronal reformations (A,B) through the neck show a curvilinear FB extending from the region of the left tonsillar fossa into the parapharyngeal space (white arrows). There is associated surrounding soft tissue inflammatory thickening (black asterisk). A few small surrounding lymph nodes are also seen (black arrows in B).

In certain cases, the FB may penetrate the mucosal lining and extend into the deep spaces of the neck, with resultant abscess formation [Figure 1]. Rare complications like internal carotid artery puncture,[8] internal jugular vein thrombophlebitis, brachial plexus injury and lodgment within the thyroid gland have all been reported.[9,10]

**Figure 2:**
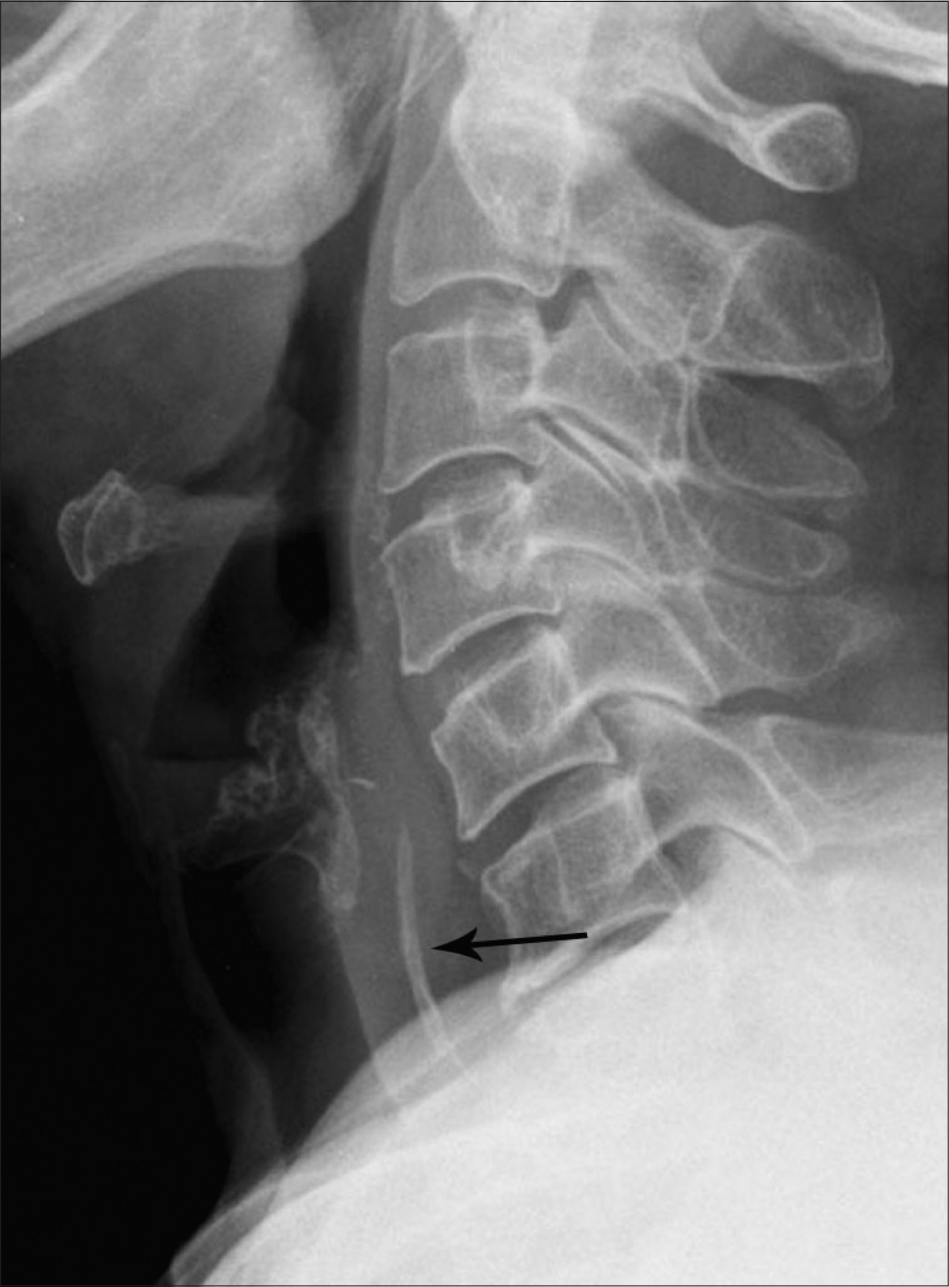
Lateral radiograph of the neck of an elderly patient shows a linear FB within the hypopharynx at the C5-6 level (black arrow). No evidence is seen of any thickening of the prevertebral soft tissues or cervical emphysema

**Figure 3:**
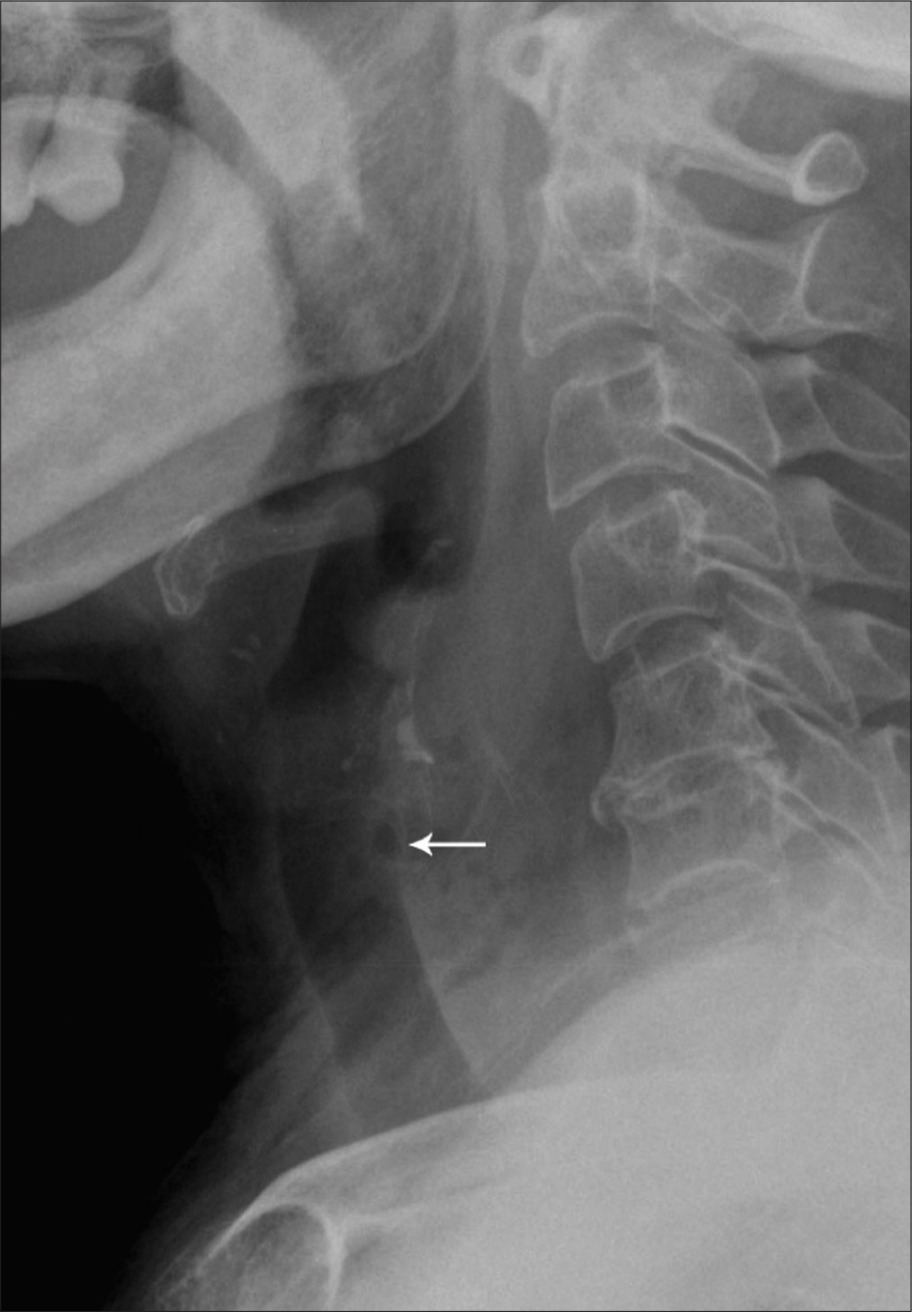
Lateral radiograph of a patient shows widening of the prevertebral space, with small air locules within (arrow), findings consistent with a retropharyngeal abscess. A noncontrast CT scan (not shown) revealed the presence of an underlying FB, which is obscured on the radiograph by the surrounding soft tissue

When the FB is in the upper aerodigestive tract, a plain radiograph is usually the initial and most commonly ordered radiological investigation. It may show the FB as a linear calcific structure, in which case no further imaging is usually required [Figure 2]. However, in certain cases, the FB may not be seen, especially if it is impacted at the level of the tonsils or at the base of the tongue. An FB impacted within the cervical esophagus on the other hand is more likely to be seen.[7] Other suspicious findings on a lateral radiograph include widening of the prevertebral soft tissue and the presence of retropharyngeal air [Figure 3]. However, plain radiography has been shown to have a sensitivity of only 32% for FB detection in the upper aerodigestive tract and esophagus.[6]

In cases where plain radiography is unsuccessful, follow-up imaging with noncontrast CT scan may be used to visualize the FB as the CT scan has a higher sensitivity. In addition, it helps to better define the associated complications like perforation or abscess formation. CT scan is also useful when looking for migration of the FB to a site outside the aerodigestive tract. Although a barium swallow may be performed as an inexpensive alternative, one must keep in mind that it is not as sensitive as the CT scan. Another major drawback is that barium coats the esophagus and makes subsequent esophagoscopy or examination very difficult.[11]

### Esophagus

**Figure 4 (A, B):**
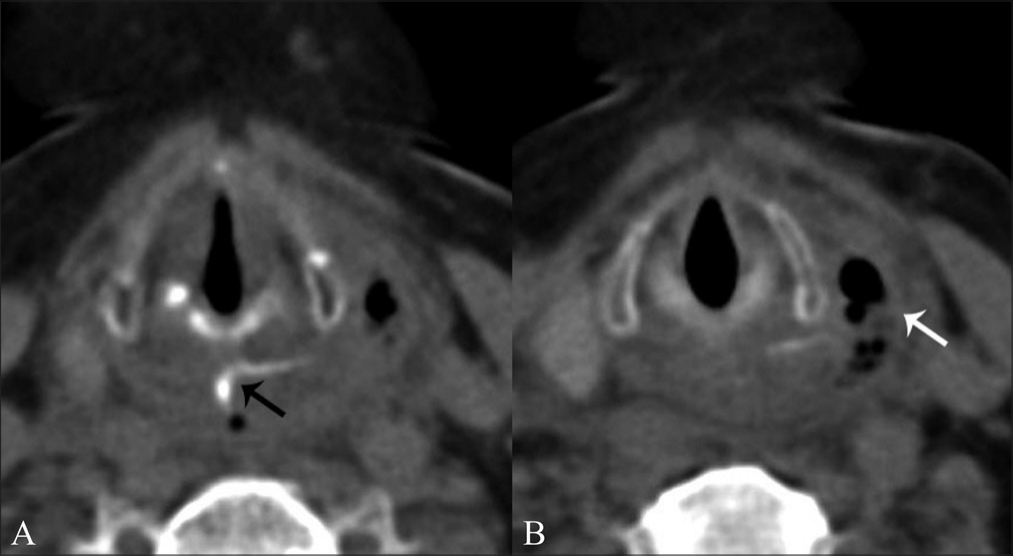
Noncontrast axial CT scans show a curvilinear FB (black arrow in A) at the level of the cricopharyngeus. There is perforation of the pharyngeal wall on the left side with a small parapharyngeal abscess (white arrow in B)

**Figure 5:**
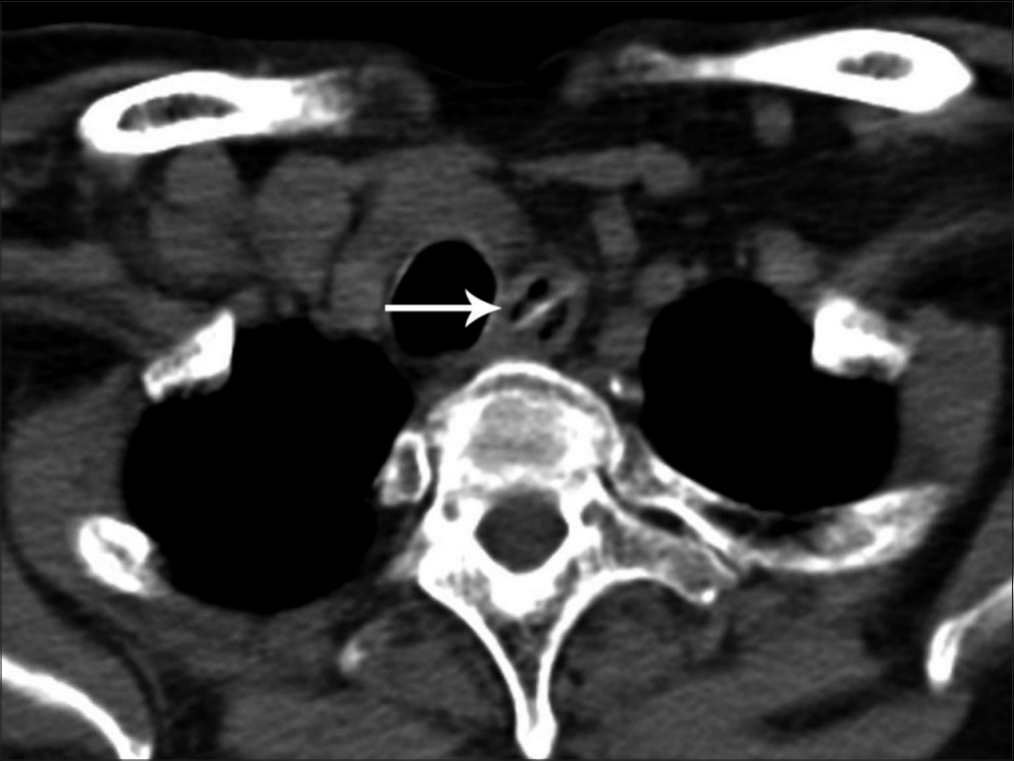
Noncontrast axial CT scan shows a linear radiodense FB (white arrow) in the upper dorsal esophagus. Note that laterally the FB is seen to extend into the wall of the esophagus. On endoscopy, it was found to be partially embedded within the wall of esophagus, with the presence of a small tear

**Figure 6 (A,B):**
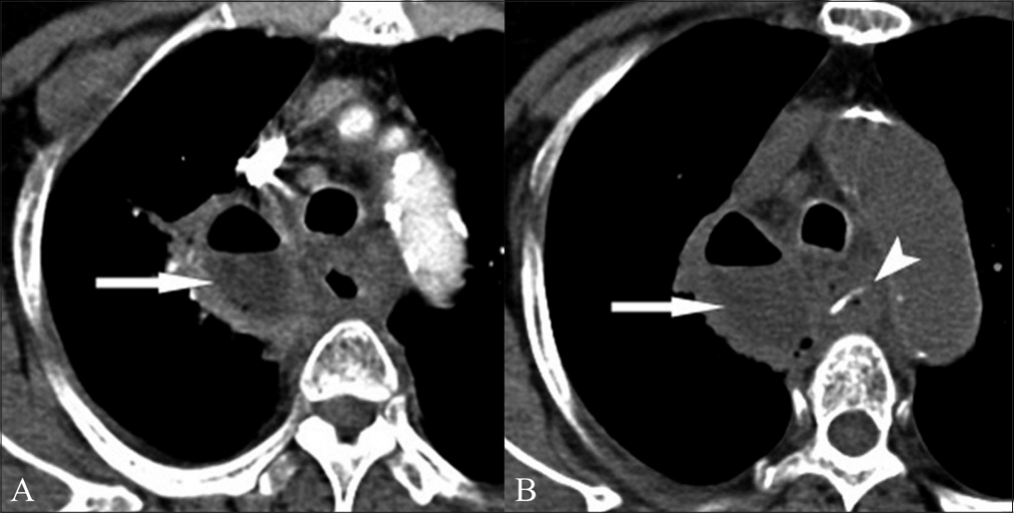
Contrast-enhanced (A) and noncontrast (B) axial CT scans show a large mediastinal abscess (arrows in both A and B) in the right paratracheal region, with associated stranding of the surrounding mediastinal fat. The esophageal wall is thickened. A linear FB (arrowhead in B) is seen within the lumen of the esophagus at the level of the arch of the esophagus

**Figure 7 (A,B):**
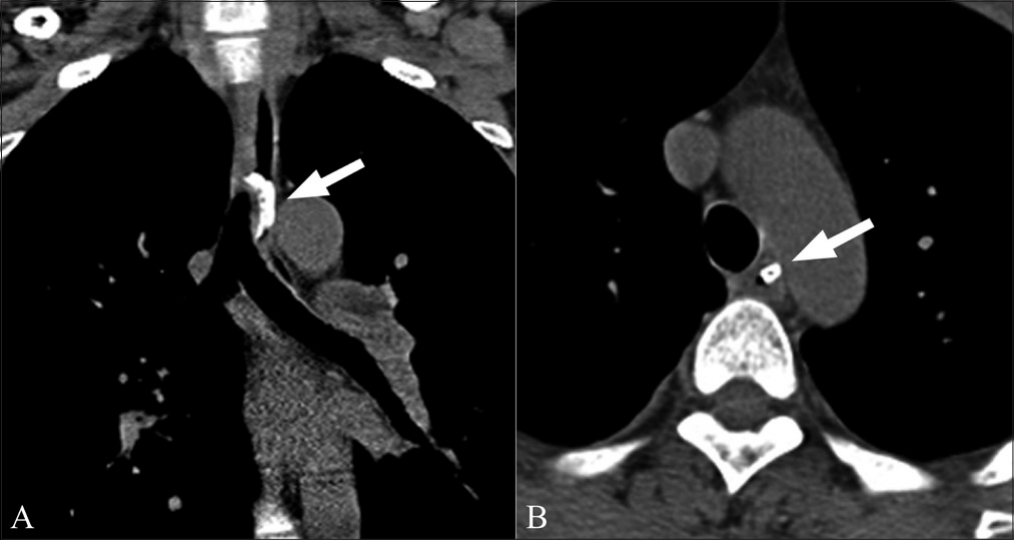
Coronal (A) and axial (B) noncontrast CT scans show a curvilinear FB at the level of the aortic arch (arrow). This is the second most common site of FB impaction within the esophagus. Note the close proximity to the aortic lumen which would partly explain the high incidence of vascular injuries within the mediastinum

Within the esophagus, the most common site of impaction is the cervical esophagus at the level of the cricopharyngeus muscle [Figure 4]. This is followed by the thoracic esophagus, where impaction is usually at the level of the aortic arch.[12] Perforation occurs in 1-4% of the cases [Figure 5]. The main causes of death however are injuries to the surrounding vascular structures or secondary suppurative complications[12] [Figure 6]. The former may manifest as an aorto-esophageal or subclavian arterio-esophageal fistula. The site of the aortic tear has been reported to be 1–5 cm from the origin of the left subclavian artery [Figure 7]. Other possible sites include the origin of the left subclavian or a right aberrant retroesophageal artery.[13]

### Abdomen and pelvis

Perforations distal to the esophagus occur in <1% of the cases[2,14,15] and are more challenging to diagnose, both clinically and radiologically. Accidentally ingested FBs may not be remembered by patients.[15] In addition, the onset of symptoms may be preceded by a time lag of months or even years, further complicating the problem.[14,16] Such patients usually present with features of peritoneal irritation, abdominal pain, bowel obstruction and/or sepsis, and the first clinical impression is often of appendicitis or diverticulitis.

Within the bowel, the most common sites of perforation are the ileum, the ileocecal junction and the rectosigmoid colon.[1–3,15,17] Acute angulations, change in direction and transition from a mobile to an immobile segment of bowel are thought to predispose to perforation by an ingested FB.[2] Rarely, the FB may perforate the duodenum, a Meckel diverticulum or the appendix.[2,18] Rare cases of a hepatic abscess with an embedded FB secondary to a duodenal perforation and a duodenocaval fistula have also been reported.[4,17]

When looking for FBs below the diaphragm, plain radiographs are generally not of sufficient diagnostic value to be used routinely as they do not show the culprit FB in most cases. This is in contrast to metallic objects and chicken bones that are invariably seen on plain radiographs.[14] An FB may not be seen for a variety of reasons. Firstly, not all FBs are sufficiently radiopaque to be seen on radiographs. In addition, the presence of fluids or large soft tissue masses or the use of a high kV exposure setting may further obscure the faint calcifications.[1,2]

Evaluation with ultrasonogram (USG) is also not always useful as the examination may be hampered by patient obesity and bowel gases. It also relies heavily on the skill of the operator. If seen, the FB may appear as a thin, linear hyperechoic structure with distal acoustic shadowing. In general, USG is more useful in thinner patients and when the perforation is relatively superficial.[2]

CT scan is the most sensitive modality when looking for an FB, and it remains the preferred investigation in such cases.[2,19] On CT scan, an FB is usually seen as a calcified linear structure surrounded by inflammatory tissue and is invariably seen if specifically looked for. In fact, the most common reason for overlooking an FB is the lack of observer awareness.[1]

**Figure 8 (A-D):**
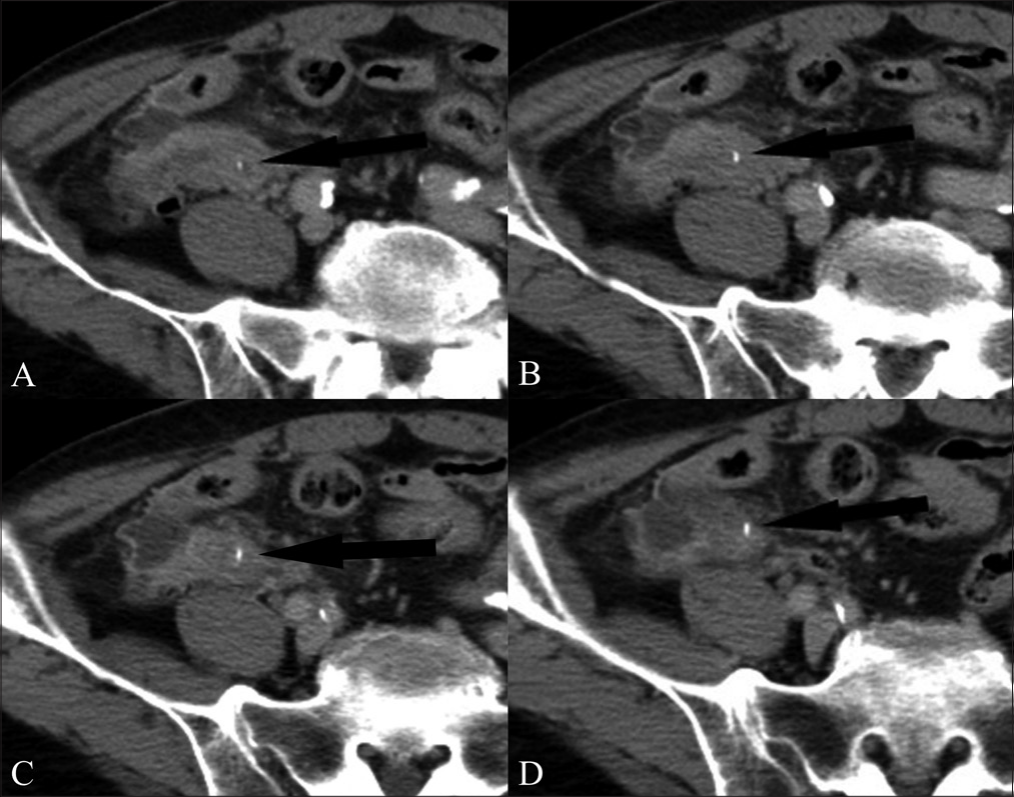
Sequential axial contrast-enhanced CT scans through the lower abdomen show a linear radiodense FB (arrow) in the distal ileum. It can be seen to traverse the thickened bowel wall. Surrounding stranding of the mesenteric fat is noted. There was no pneumoperitoneum

**Figure 9 (A, B):**
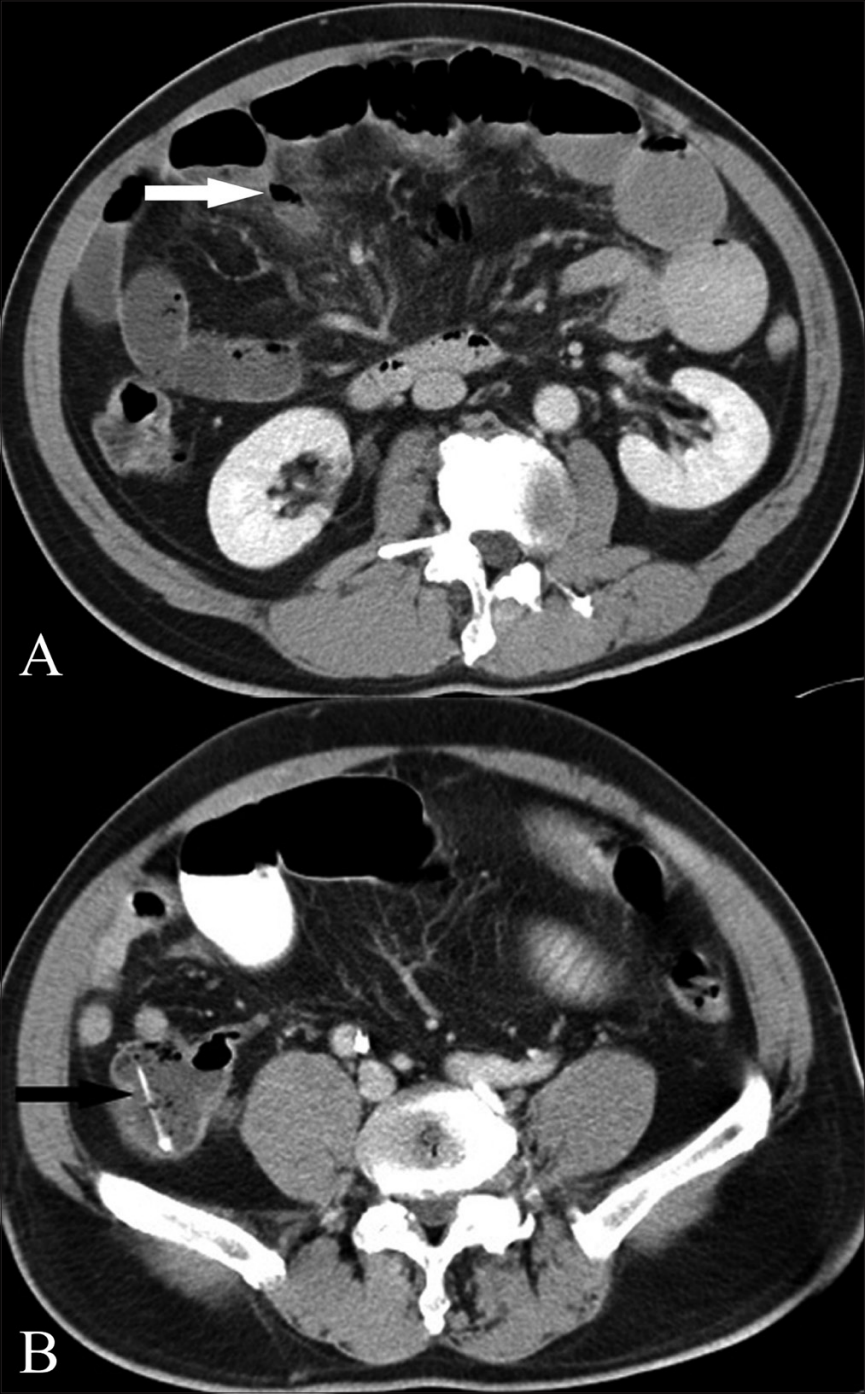
Axial contrast-enhanced CT scans show focal fat stranding and localized pneumoperitoneum in relation to the ileal loops (white arrow in A). There is associated dilatation of the bowel loops, suggesting obstruction. The FB is seen distally in the large bowel (black arrow in B), reemphasizing the importance of closely scrutinizing the bowel loops distal to the inflamed segments

Following FB ingestion, the most common complication below the diaphragm is perforation of a hollow viscus [Figure 8]. On CT scan, the region of perforation may be identified as a thickened bowel segment, with regional fatty infiltration or bowel obstruction.[1,3] Peritoneal effusions may also be present. Localized pneumoperitoneum may be seen in up to half of the patients.[2] Free peritoneal air, on the other hand, is rare as the FB is gradually impacted and the site of perforation may be sealed-off by omentum or surrounding inflammation.[3] There may be secondary bowel obstruction or abscess formation [Figure 9].

**Figure 10 (A, B):**
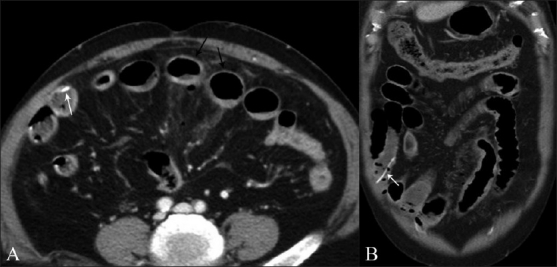
Small bowel perforation without obstruction. Axial (A) and coronal (B) contrast-enhanced CT scans show localized pneumoperitoneum and fat stranding in relation to focally thickened distal ileal loops (black arrows in A). The FB however, has since passed distally into the large bowel and can be easily overlooked on the axial images (white arrow in A). Note that the FB is fairly conspicuous on the coronal images (white arrow in B)

In some cases, the FB may migrate caudally and may be seen situated away from the site of perforation [Figure 10]. Careful scrutiny of the images in such cases usually reveals the culprit FB and it is worth looking for, especially if no other cause of bowel perforation is apparent. It is also important to remember that there may be more than one site of perforation.

**Figure 11 (A, B):**
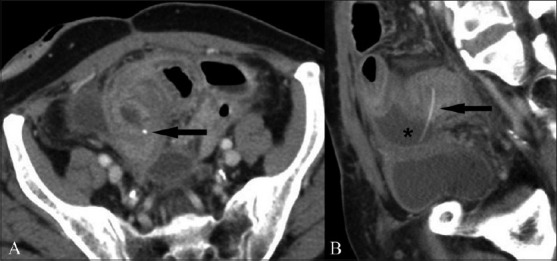
Axial (A) contrast-enhanced CT scan shows a small hyperdensity, which may be easily overlooked or confused with a vessel (black arrow in A), which however is very well appreciated in the sagittal reformatted image (arrow in B) penetrating through the bowel wall. Associated segmental bowel wall thickening is seen with presence of a small loculated collection (asterisk in B)

**Figure 12 (A-C):**
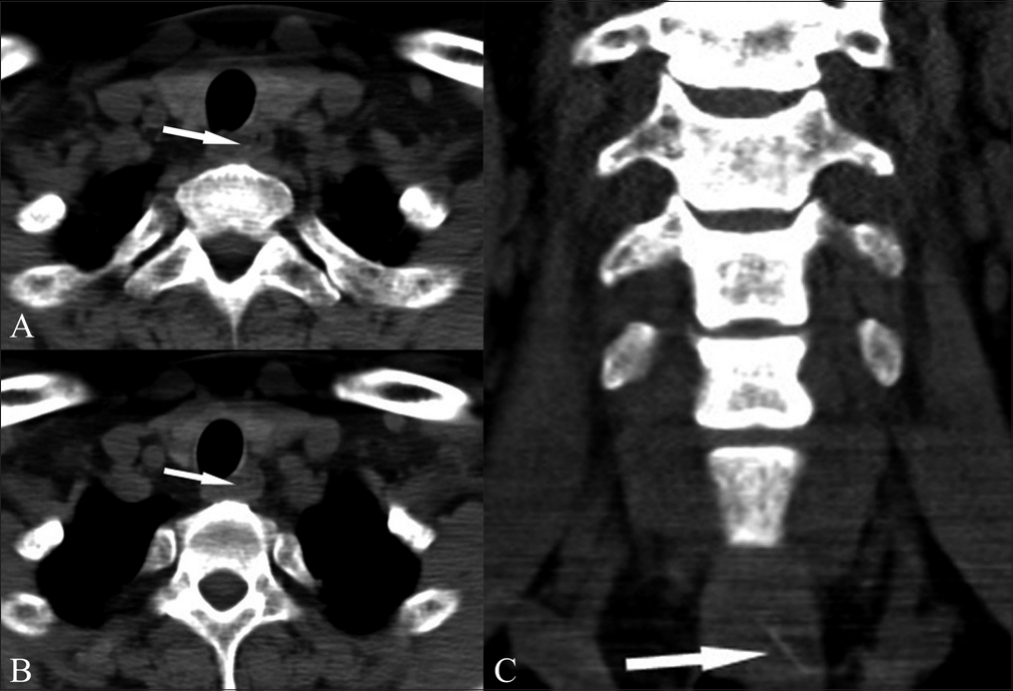
Non-contrast axial (A,B) CT scans show a questionable hyperdensity within the esophageal lumen, at the level of the thoracic inlet (arrows). The coronal reconstructed image however clearly shows the presence of a fine linear FB (arrow in C). Coronal images are in general more useful in cases of FB since the bone is usually oriented orthogonally to the acquired axial images

Despite the clear superiority of CT scan over plain radiography and USG, there are certain potential pitfalls that must be kept in mind. The faint calcification of FB may be obscured by oral contrast.[1] In such cases, delayed or repeat scanning without oral contrast may be more useful. In cases where intravenous contrast has been given, the FB may mimic a small blood vessel[1,14] and can be easily overlooked [Figure 11]. Another potential limitation of CT scan is slice thickness. It is generally agreed that thinner reconstructions (3 mm/1.5 mm) are better than thick conventional sections (5 mm/8 mm).[1,2] Finally, the orientation of the FB with respect to the axial scans may affect viewer perception. In such cases, coronal or sagittal reconstructions may be especially useful [Figure 12].

FB perforations may also mimic neoplastic conditions. Sporadic cases of an FB masquerading on imaging as a tongue malignancy,[20] esophageal mass,[21] gastric submucosal tumor[19] and even a locally advanced pancreatic tumor[14] have been reported. This is partly due to the intense inflammatory reaction induced and in part due to the lack of observer awareness about the imaging appearance of the FB. In virtually all these cases, a linear, calcified hyperdensity consistent with an FB was seen either at the time of scanning or on retrospective analysis.

## Conclusion

Although in most cases the ingested FB uneventfully passes through the GI tract, it has the potential to cause a variety of complications. Because the history may not always be forthcoming and because patients may present with nonspecific symptoms, FBs are relatively easy to overlook radiologically. A high index of suspicion and a diligent search for the FB are usually rewarding in such cases.
